# Role of TORS as De-Escalation Strategy in HPV-Related Oropharyngeal Cancer, What We Need to Know

**DOI:** 10.3390/healthcare12101014

**Published:** 2024-05-14

**Authors:** Gabriele Molteni, Sara Bassani, Athena Eliana Arsie, Erica Zampieri, Giuditta Mannelli, Ester Orlandi, Paolo Bossi, Armando De Virgilio

**Affiliations:** 1 Department of Otolaryngology-Head and Neck Surgery, IRCCS Azienda Ospedaliero-Universitaria di Bologna, 40138 Bologna, Italy; 2 Department of Medical and Surgical Sciences, Alma Mater Studiorum-University of Bologna, 40126 Bologna, Italy; 3 Otolaryngology-Head and Neck Surgery Department, University of Verona, 37129 Verona, Italy; athenaeliana.arsie@studenti.univr.it (A.E.A.);; 4 Department of Experimental and Clinical Medicine, University of Florence, 50134 Firenze, Italy; 5 Department of Clinical, Surgical, Diagnostic, and Pediatric Sciences, University of Pavia, 27100 Pavia, Italy; ester.orlandi@cnao.it; 6 Clinical Department, National Center for Oncological Hadrontherapy (Fondazione CNAO), 27100 Pavia, Italy; 7 Department of Biomedical Sciences, Humanitas University, Via Rita Levi Montalcini 4, 20072 Pieve Emanuele, Italy; paolo.bossi@hunimed.eu; 8 IRCCS Humanitas Research Hospital, Via Manzoni 56, 20089 Rozzano, Italy; 9 Otorhinolaryngology Unit, IRCCS Humanitas Research Hospital, Via Manzoni 56, 20089 Rozzano, Italy

**Keywords:** TORS, HPV, de-escalation, oropharynx

## Abstract

Human papillomavirus (HPV)-related oropharyngeal squamous cell carcinoma (OPSCC) presents unique challenges and opportunities for treatment, particularly regarding de-escalation strategies to reduce treatment morbidity without compromising oncological outcomes. This paper examines the role of Transoral Robotic Surgery (TORS) as a de-escalation strategy in managing HPV-related OPSCC. We conducted a comprehensive literature review from January 2010 to June 2023, focusing on studies exploring TORS outcomes in patients with HPV-positive OPSCC. These findings highlight TORS’s potential to reduce the need for adjuvant therapy, thereby minimizing treatment-related side effects while maintaining high rates of oncological control. TORS offers advantages such as precise tumor resection and the ability to obtain accurate pathological staging, which can guide the tailoring of adjuvant treatments. Some clinical trials provide evidence supporting the use of TORS in specific patient populations. The MC1273 trial demonstrated promising outcomes with lower doses of adjuvant radiotherapy (RT) following TORS, showing high locoregional tumor control rates and favorable survival outcomes with minimal side effects. ECOG 3311 evaluated upfront TORS followed by histopathologically directed adjuvant therapy, revealing good oncological and functional outcomes, particularly in intermediate-risk patients. The SIRS trial emphasized the benefits of upfront surgery with neck dissection followed by de-escalated RT in patients with favorable survival and excellent functional outcomes. At the same time, the PATHOS trial examined the impact of risk-adapted adjuvant treatment on functional outcomes and survival. The ongoing ADEPT trial investigates reduced-dose adjuvant RT, and the DART-HPV study aims to compare standard adjuvant chemoradiotherapy (CRT) with a reduced dose of adjuvant RT in HPV-positive OPSCC patients. These trials collectively underscore the potential of TORS in facilitating treatment de-escalation while maintaining favorable oncological and functional outcomes in selected patients with HPV-related OPSCC. The aim of this scoping review is to discuss the challenges of risk stratification, the importance of HPV status determination, and the implications of smoking on treatment outcomes. It also explores the evolving criteria for adjuvant therapy following TORS, focusing on reducing radiation dosage and volume without compromising treatment efficacy. In conclusion, TORS emerges as a viable upfront treatment option for carefully selected patients with HPV-positive OPSCC, offering a pathway toward treatment de-escalation. However, selecting the optimal candidate for TORS-based de-escalation strategies is crucial to fully leverage the benefits of treatment de-intensification.

## 1. Introduction

Traditionally, oropharyngeal squamous cell carcinoma (OPSCC) has been associated with habits like alcohol consumption and tobacco use. However, in many high-income countries, there has been a decline in smoking rates over the past two decades, leading to a decrease in the overall incidence of HNSCC. Despite this trend, the prevalence of OPSCC has risen due to another significant risk factor: infection with carcinogenic strains of human papillomavirus (HPV). This viral infection has become increasingly recognized as a critical driver behind the increased incidence of OPSCC during the same period [1].

Available evidence indicates a favorable prognosis for patients with HPV-related OPSCC when compared to those who are HPV-unrelated. This enhanced outcome is mainly due to the heightened responsiveness of HPV-positive tumors to radiation and chemotherapy treatments [2]. Traditionally, treatment protocols for both HPV-positive and HPV-negative OPSCC have been similar despite their different clinical outcomes [3]. Expanding on the unique biological and clinical characteristics of HPV-positive versus HPV-negative OPSCC can provide a more precise rationale for tailored treatment approaches, such as TORS as a de-escalation strategy, which is particularly beneficial for HPV-positive cases. This approach clarifies the specialized needs of these patients and underscores the significance of such distinctions in the broader context of head and neck oncology [3].

Traditionally, HPV-related OPSCC was primarily managed through radiotherapy (RT), administered as intensity-modulated RT (IMRT) and chemotherapy (CT). Recent clinical trials have explored various strategies for less aggressive treatment approaches. These strategies can be categorized into four main types [4]:

- Modification of chemoradiation (CRT) protocols: this approach involves either reducing the dosage or replacing cisplatin with targeted drugs like cetuximab, used alongside RT.
- Sequential therapy with induction CT: patients first receive induction CT. If they show a favorable response to CT, they might then undergo a lower total dose of RT or less extensive target volume compared to the standard RT approach, or they might receive conservative surgery alone.
- RT as an exclusive approach: some studies are investigating the effectiveness of using only RT, with either a standard or reduced dose, in place of the more conventional CRT treatment.
- Minimally invasive transoral surgery, such as transoral robotic surgery (TORS) or transoral laser microsurgery (TLM): these surgical approaches are gaining prominence, particularly for early-stage HPV-related OPSCC (T1-T2). They offer improved visualization and precise control, facilitating thorough pathological staging through resection with clear margins. The detailed insights obtained from surgical staging may enable more personalized postoperative treatments, particularly in HPV-positive patients, potentially minimizing the need for intensive follow-up therapies and associated complications. Furthermore, in cases where HPV-positive lymph node cervical metastases are present without a detectable primary tumor (CUP), TORS plays a crucial role in identifying the primary tumor site, allowing for a reduction in RT field or dosage to the oropharyngeal mucosa. The advent of de-escalation strategies represents a significant evolution in the management of OPSCC. With its ability to provide precise surgical staging and facilitate targeted therapy, TORS aligns with the goals of de-escalation strategies by enabling tailored treatment plans that optimize outcomes while minimizing treatment-related toxicities [5].

In the context of HPV-related OPSCC, the decision to opt for surgical methods like TORS over non-surgical treatments such as CRT should consider the possibility of lessening the intensity of adjuvant therapy in order to reduce the burden of toxicity. This article aims to provide insights into the decision-making process regarding the use of TORS and the potential for reduced postoperative adjuvant treatment in OPSCC, enhancing functional outcomes and quality of life.

## 2. Materials and Methods

### Search Strategy

We strictly adhered to the Preferred Reporting Items for Systematic Reviews and Meta-Analyses (PRISMA) [6] guidelines.

For our search strategy, we conducted a thorough systematic search of articles published between January 2010 and June 2023 in the PubMed and Web of Science databases with the combined query: (“HPV-associated oropharyngeal cancer” OR “HPV-related oropharyngeal carcinoma” OR “OPSCC”) AND (“treatment” OR “therapeutic approaches” OR “management” OR “surgical options” OR “radiation therapy” OR “chemotherapy” OR “de-escalation” OR “intensity reduction” OR “outcomes” OR “quality of life” OR “TORS”). The choice of this time frame is because, to our knowledge, the concept of de-escalation in the management of OPSCC has gained ground since 2010 [7], subsequently developing with new studies.

Subsequently, the full text of relevant studies was screened for final selection. All studies identified by the initial literature search were reviewed independently by two authors. All titles and abstracts were assessed. Our selection criteria for studies on OPSCC included articles published between January 2010 and June 2023, featuring patients with confirmed OPSCC diagnoses based on histopathological examination, with a precise determination of HPV or p16 status on surgical specimens, who underwent TORS. We excluded duplicate publications, reviews, case series with fewer than ten patients, book chapters, case reports, and poster presentations. Our focus was explicitly on OPSCC studies, and we excluded those discussing other histologies or surgical treatments aside from TORS. Additionally, studies lacking clarity on HPV or p16 status or not published in English were excluded from our analysis.

**Figure 1:**
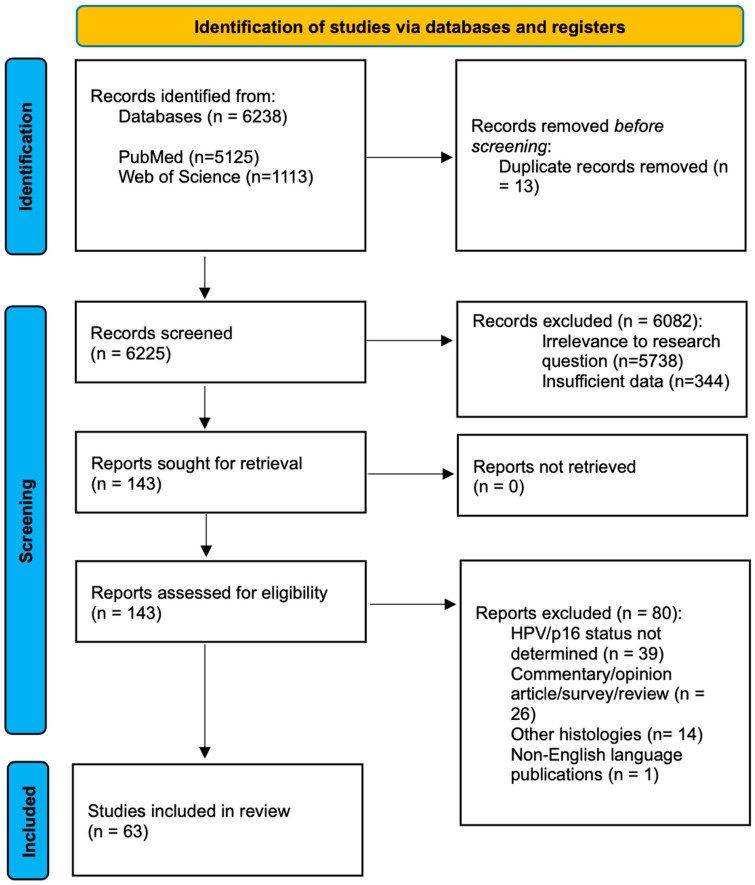
PRISMA.

The selection process is summarized in Figure 1.

## 3. Results

Following the screening process, we assessed the abstracts and reviewed the full texts of the articles. Our selection led to the inclusion of 63 relevant articles, and we compiled essential information for each article, including authorship, country, publication year, sample size, and key findings. The articles are summarized in Table 1.

Through the examination of the chosen articles, we identified key areas that raise questions regarding the assessment of HPV status, the patients’ selection, the guidelines for endorsing or discouraging the use of TORS, and the potential roles of TORS in the context of de-escalation strategies.

## 4. Discussion

### 4.1. HPV Status Determination

Identifying HPV status is crucial in HPV-associated OPSCC, as it indicates a unique disease type with a specific molecular background. The immunohistochemical detection of p16, a key marker for HPV positivity, is of paramount importance in this context. The overexpression of p16, often a result of HPV types 16 and 18 disrupting p53 and pRB through their oncoproteins E6 and E7, serves as a proxy for HPV involvement in these cancers. The threshold for determining p16+ by immunohistochemistry is a nuclear expression of ≥+2/+3 with a distribution of >75% of the neoplasm [71].

Patients with p16-positive OPSCC exhibit markedly different prognoses compared to those with p16 negative OPSCC. Younger patients with p16-positive OPSCC generally show better treatment responses. For instance, a 5-year overall survival rate for stage IV p16 positive OPSCC is about 70%, significantly higher than the 30% for p16 negative cases. The 8th edition of the AJCC Cancer Staging Manual reflects these differences, offering distinct staging criteria for p16-positive and -negative OPSCC [72].

Another critical concern is assessing p16 expression and HPV DNA presence in OPSCC cases. The gold standard approach involves examining both factors since some cases may exhibit discordance between them. Notably, patients who are either p16- or HPV-positive tend to have a more favorable prognosis compared to those who are negative for both. However, those who are positive for both p16 and HPV show an even better prognosis, indicating an intermediate prognosis for patients with single positivity [73]. These highlight the need to include ‘real’ HPV+ OPSCC in future studies, and especially in a de-escalation setting.

### 4.2. Risk Determination

Several prognostic models have emerged, factoring in p16 status, smoking history, and other clinical parameters. Ang et al. [7] identified three risk groups based on these factors, observing a significant difference in overall-survival (OS) between non-smoker p16-positive patients and p16-positive patients with a smoking history of >10 pack–year and N2–N3 disease. However, their classification was limited to a specific trial population. Deschuymer et al. [74] introduced a new risk group classification for p16-positive OPSCC, focusing on the 8th staging edition, comorbidities, and smoking history. They identified a low-risk group of patients, defined as stage I, never smokers or smokers with less than 10 pack–year smoking history and low comorbidity, that showed an excellent prognosis and could benefit from de-escalation trials. Rietbergen proposed another risk model based on an unselected European cohort, considering comorbidities along with p16 status and N-stage [75]. Lassen et al. [76] further emphasized the impact of smoking on survival in p16-positive OPSCC patients, suggesting that active smokers and >30 pack–year history patients might not be ideal candidates for de-escalation trials due to decreased radiotherapy efficacy.

One key factor that could reduce the effectiveness of RT in individuals who actively smoke is tumor hypoxia. This is because the mechanism of RT largely relies on generating free radicals, a process heavily dependent on the presence and level of oxygen within the tumor. Smoking is known to lower the effectiveness of hemoglobin and the delivery of oxygen to tissues, including tumor cells [77]. However, current research on the influence of smoking in patients undergoing surgical treatments remains limited.

A study conducted by Roden et al. [78], primarily focusing on patients in clinical stage I OPSSC, found that smoking did not significantly affect recurrence-free survival (RFS), OS, or disease-specific survival (DSS). These patients underwent TORS as an initial treatment, followed by pathology-guided adjuvant therapy. The study observed that smokers were more likely to present with extra capsular spreading (ECS) and positive margins, potentially leading to more intensive treatment. However, these factors did not seem to adversely affect their prognosis. The authors of the study hypothesize that the surgical removal of tumor bulk might enhance the effectiveness of RT in such cases where tissue oxygenation is compromised due to smoking. This does not seem to adversely affect local tumor control. Therefore, they suggest that smokers, especially those with early-stage HPV-related OPSCC falling into the intermediate risk category, should not be automatically excluded from TORS-based de-escalation clinical trials.

In this regard, a recent study added information on the predictive role of tumor hypoxia in response to radiation and the possibility of modulating the dose of radiotherapy. The use of functional hypoxia imaging allowed for a drastic reduction in the dose of radiation to 30 Gy without hampering treatment efficacy and with advantages in acute and late toxicities [79].

### 4.3. TORS Indication

TORS is emerging as a preferred treatment for HPV-positive OPSCC, offering advantages like a reduced need for reconstructive surgery and shorter operation times compared to traditional methods. TORS follows principles of minimal invasiveness and patient-specific factors like anatomy, and prior treatments significantly influence its feasibility [80,81,82]. Preoperative evaluations, including the 8Ts of endoscopic access (teeth, trismus, transverse mandibular dimensions, tori, tongue, tilt, prior RT, and tumor) [83], are also crucial in determining the suitability of TORS for a patient.

Obviously, in addition to the characteristics related to the patient, there are also characteristics related to the tumor that must be taken into consideration. In particular, three categories of contraindications to TORS related to the tumor have been identified: vascular, functional, and oncological [82].

Regarding vascular factors, TORS is not recommended in cases where a tonsillar malignancy is present alongside a retropharyngeal carotid artery, or if the tumor is located centrally at the tongue base or in the vallecula. Additionally, the proximity of the tumor to vital vascular structures such as the carotid bulb or internal carotid artery, or carotid artery encasement by the tumor or metastatic neck nodes, also rules out the use of TORS. From a functional perspective, TORS is contraindicated if the tumor removal would necessitate the excision of more than half of the deep musculature of the tongue base or the posterior pharyngeal wall. Similarly, TORS is not suitable in case of both tongue base and entire epiglottis removal. On an oncological level, there are several scenarios where TORS appears inappropriate: advanced cancers (T4b), unresectable neck disease, or in the case of multiple and multiple-site metastases. Even trismus caused by the tumor, the prevertebral fascia or the mandible or hyoid bones involvement, the lateral neck’s soft tissues tumor extension, or Eustachian tube involvement do preclude the use of TORS [84].

**Figure 2:**
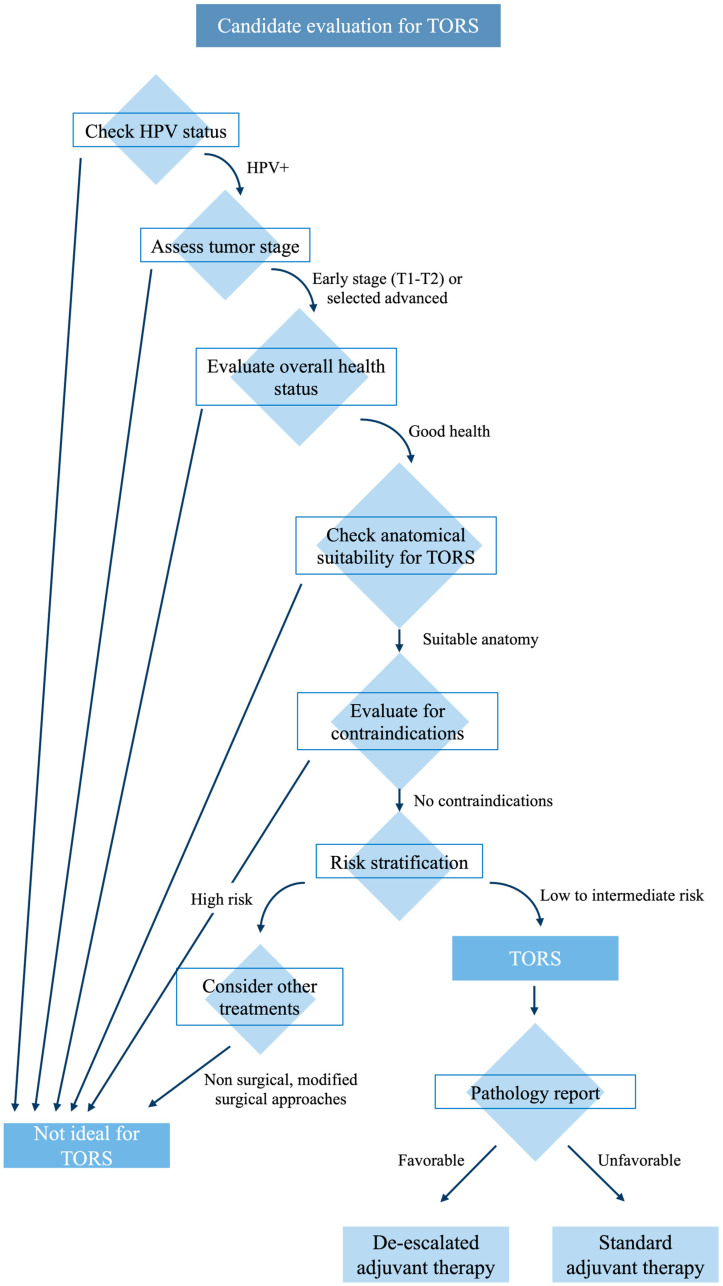
Candidate selection summary.

Despite the evolving role of TORS, the optimal postoperative treatment for HPV-positive OPSCC following this surgery remains to be defined (Figure 2).

### 4.4. Role of TORS as a De-Escalation Strategy

Over the last decade, TORS has been increasingly used in the treatment of HPV-related OPSCC. In a de-intensification treatment scenario, the histopathological data obtained from initial surgical procedures might provide an opportunity to lower the adverse side effects associated with non-surgical adjuvant treatments, both in the short and long term.

Some clinical trials evaluate de-escalation after TORS.

The MC1273 trial was a phase II study that focused on using a lower dose of adjuvant RT after TORS/TLM. It included patients with HPV-positive OPSCC and a smoking history of less than 10 pack–year and had undergone a surgical removal of tumors with clear margins [85].

Patients at intermediate risk received 30 Gy of adjuvant RT over 2 weeks, while those with ECS on their final pathology report received 36 Gy. The trial showed promising results, with a 2-year locoregional tumor control rate of 96.2%, a progression-free survival rate of 91.1%, and an overall survival rate of 98.7%. The occurrence of severe side effects before RT and at 1- and 2-year follow-ups was low, at 2.5%, 0%, and 0%, respectively. Additionally, there was a slight improvement in swallowing function between the time before RT and 12 months after RT [85].

ECOG 3311 (NCT01898494) took into consideration HPV+ OPSCC in stages III–IVb, the treatment performed was upfront TORS followed by histopathologically directed adjuvant therapy in order to identify which selected patients could benefit from de-escalated RT (observation/50 vs. 60 Gy/66 Gy with weekly cisplatin). This clinical trial showed that primary TORS and reduced postoperative RT result in good oncological outcome and favorable functional outcomes in intermediate-risk HPV+ OPSCC, even if the highest difference in quality of life and swallowing were identified when comparing the single modality or the double modality with the tri-modality treatment [86].

SIRS Trial (NCT02072148) took into consideration only T1 and T2 categories. After TORS, patients were assigned to group 1 (no poor risk features; surveillance), group 2 (intermediate pathological risk factors [perineural invasion, lymphovascular invasion]; 50-Gy radiotherapy), or group 3 (poor prognostic pathological factors [ECS, more than three positive lymph nodes and positive margins]; concurrent 56-Gy chemoradiotherapy with weekly cisplatin). The findings suggest that performing upfront surgery with neck dissection, followed by a de-escalated RT, is associated with favorable survival outcomes and excellent functional outcomes in patients with T1–2, N1 stage p16+ OPSCC [87].

Clinical trial PATHOS (NCT02215265) is another phase II trial examining the impact of a transoral laser resection of tumors followed by risk-adapted adjuvant treatment, on functional outcomes and survival. Patients undergo TLM/TORS resection of tumors, and are then randomized to reduced dose RT or standard dose RT or to concurrent chemoradiation or RT alone, according to pathological risk factors [88].

ADEPT trial (NCT01687413) is currently investigating reduced dose-adjuvant RT and removal of chemotherapy from the adjuvant regimen of patients with ECS on final pathology [89].

The DART-HPV study (NCT02908477) is presently in the process of enrolling participants at the Mayo Clinic. In this study, patients with HPV-positive OPSCC who have undergone transoral resection and meet the criteria for adjuvant treatment are randomly assigned to either receive standard adjuvant CRT or docetaxel in combination with a reduced dose of adjuvant RT (30 Gy administered over two weeks).

### 4.5. TORS as a De-Escalation Strategy for cT1-cT2

Currently, there is a lack of consensus regarding the optimal supplementary treatment following upfront TORS for patients with stage I OPSCC who test positive for p16. The primary areas of uncertainty in the postoperative phase focused on two key aspects: the necessity for concurrent CT when dealing with ECS and positive margins and determining the appropriate RT dose and volume size in relation to the extent of surgical intervention. [90]

The criteria for recommending adjuvant RT are derived from the guidelines established by the National Comprehensive Cancer Network (NCCN) [91]. These criteria include: ECS, close or positive margins, pT3 or pT4 primary tumor, one positive lymph node measuring >3 cm or multiple positive nodes, nodal involvement in levels IV or V, perineural invasion, vascular invasion, and lymphatic invasion [92].

In cases of margin positivity or ECS, combining CT with RT is highly recommended [93].

Some studies have shown that TORS yields positive oncological and functional results in the management of early-stage HPV-related OPSCC [8,11,21,22,26,27,34,35,38,41,44,48,51,61,64,69]. In clinical practice, many patients diagnosed with cT1 and cT2 HPV-related OPSCC often undergo upfront TORS. The indication for adjuvant RT with or without concomitant CT follows the abovementioned criteria, considering the number and types (major versus minor) of risk factors. The standard approach for RT involves targeting the primary tumor and lymph node regions at risk, delivering a dosage ranging from 50 to 66 Gy with conventional fractionation.

When it comes to strategies for de-intensifying adjuvant RT, two main options are being explored:

1. Reducing the RT total dose;
2. Reducing the extension of RT target volumes. One noteworthy strategy being investigated involves omitting adjuvant RT to the primary tumor site in cases of early T stages [89].

TORS enables precise intraoperative margin assessment, leading to a high rate of margin-negative resections and consequently low local recurrence rates, especially in early T-stage tumors [13,42,46,57,64]. The potential benefit of excluding the primary tumor site from the radiation field lies in minimizing local toxicity in a critical anatomical area, thus resulting in reduced treatment-related morbidity.

In a 2016 study, it was demonstrated that excluding RT treatment to the primary tumor site in margin-negative resected T1–T2 p16-positive OPSCC did not result in a significant compromise in terms of local control [94]. Among 202 T1–T2 patients, 92 did not receive planned RT to the primary tumor bed, with 48 of them not receiving any adjuvant treatment and 44 receiving RT only to the ipsilateral neck [94]. This group showed a local recurrence rate of 3%, compared to 0% in patients who received radiation to the primary site [94]. Furthermore, patients who did not receive planned RT to the primary site exhibited superior preservation of the swallowing function, with a temporary gastrostomy rate of 6.5%, in contrast to 41% in patients who received radiation to the primary site [94].

The first prospective single-arm phase II clinical trial published in 2020 from University of Pennsylvania (NCT02159703) yielded different outcomes when assessing the safety and effectiveness of focusing RT solely on the neck, while excluding treatment for the primary tumor site. This study included 60 patients diagnosed with stage pT1–pT2, N1–3 p16-positive OPSCC who had undergone TORS and selective neck dissection (SND). All these patients exhibited favorable features at the primary site, including negative surgical margins (≥2 mm), the absence of perineural invasion, and no lymphovascular invasion. Adjuvant RT +/− CT was administered based on lymph node involvement, including patients with extranodal extension (ENE). Target volumes (TV) for RT were defined as follows: TV1 included the ipsilateral lymph node levels II, III, and IV and any other involved lymph node level; TV2 generally included the ipsilateral level V, lateral retropharyngeal nodal stations, and the contralateral level II, III, and IV; TV3 included areas of pathological ENE if applicable. Prescriptions for the different neck TVs were 60 Gy, 54 Gy, and 63–66 Gy, respectively, in 30–33 treatment fractions. RT target volumes included all study patients’ selective nodal regions of the bilateral neck. The primary tumor site was defined as the TORS operational bed and was contoured as an avoidance structure for RT planning.

Despite this reduction in radiation dosage to the different risk areas in the neck and the primary tumor site avoidance in radiation, the study reported an excellent 2-year local control rate of 98.3% and a recurrence-free survival rate of 97.9%. Regarding adverse effects, only 3.3% of patients required a temporary feeding tube, and there was an extremely low incidence (3.3%) of soft tissue necrosis in the operative bed at the primary tumor site [61]. However, it should be observed that, due to RT planning, the mean ‘unwanted’ dose administered to the primary surgical bed was 36 Gy which is a dose equal or superior to those administered in aggressive de-escalation trials such as the MC1273 and MC1675 [85].

Additionally, another observational study conducted at the Mayo Clinic (NCT02736786) is investigating the clinical and functional outcomes of mucosal sparing proton beam therapy in patients with resected T1–T2 p16-positive oropharyngeal tumors characterized by negative margins and the absence of perineural invasion and lymphovascular invasion at the primary site [95].

This strategy to test an RT volume de-escalation approach is highly intriguing. Proton therapy offers improved tumor conformity compared to conventional photon-based radiotherapy, allowing for an accurate evaluation of volume de-escalation efficacy. It eliminates biases related to low and intermediate doses, which may control microscopic disease. Due to the proximity of the highest nodal station to the oropharyngeal mucosa, omitting low to intermediate doses—which is impossible with high conformal radiotherapy (i.e., IMRT)—could provide valuable insights into the efficacy of not irradiating the primary tumor bed after TORS [96].

### 4.6. TORS as a De-Escalation Strategy for cT3–cT4

Although TORS is often used for tumors at lower T stages, it has been employed in cases with advanced cervical disease, where it serves as the initial treatment followed by adjuvant RT and possibly CT. The existing literature presents promising data concerning oncological outcomes.

White et al. conducted a review involving 89 patients, 65% of whom had either T3–T4 tumors or N2–N3 disease [97]. This study showed that 92% of patients underwent TORS as their primary treatment, resulting in an overall 2-year survival rate of 89.3% [97]. Other studies reported comparable results [98,99]. In addition to the oncological benefits, they were employing TORS as the first-line treatment, and this offers advantages such as the ability for pathological analysis, which can lead to the upstaging or downstaging of the patient’s disease. This, in turn, may allow for a reduction in radiation doses and the potential avoidance of CT. Furthermore, the utilization of TORS offers the potential to mitigate the risk of positive margins, consequently enhancing patient survival outcomes. [37].

Hurtuk et al. reviewed 64 patients who underwent TORS, with 68.4% classified as N2–N3. The analysis of pathological specimens resulted in CT avoidance in 34% of patients with stage III/IV tumors [100]. However, this strategy should be used with caution because there is a risk involved in using a trimodality approach, which has been linked to a significant risk of acute and late toxicities that can negatively influence the quality of life [86].

In a study by Lukens et al., a 28% rate of late soft tissue necrosis was reported among patients with locally advanced oropharyngeal carcinoma who underwent treatment with TORS followed by postoperative RT. Tonsillar location, the depth of resection, radiation dose to the surgical bed, and severe mucositis were identified as independent risk factors, prompting the authors to carefully avoid a radiation dose exceeding 2 Gy per day to the surgical bed [101].

Another emerging field in this regard is the combination of neoadjuvant chemotherapy (NCT) followed by TORS. Sadeghi et al. published a prospective cohort of patients with HPV+ locoregional advanced OPSCC undergoing NCT + transoral surgery, which was compared to a historical cohort of patients undergoing CRT. The NCT + surgery group demonstrated superior DSS and disease-free survival (DFS) compared to the CRT group, with a lower incidence of severe treatment-related toxicity and feeding tube dependence [102,103].

Costantino et al. at Yonsei University, Seoul, Korea, proposed a treatment protocol consisting of NCT, including cisplatin and itanium silicate (TS-1), administered over several cycles in patients with locoregionally advanced OPSCC. About 70% of patients were p16+. TORS was performed after assessing the tumor response to NCT. The surgical approach aims to achieve a complete resection guided by pre-NCT assessments, with subsequent pathological evaluation guiding the need for adjuvant treatments. The primary tumor site showed a pathological complete response in 32.8% of patients, while regional lymph nodes exhibited a complete response in 43%. The estimated DFS rates at 1 and 3 years were 86.6% and 81.4%, respectively, with DSS rates at 1 and 3 years of 96.7% and 92.6% [20,104,105]. However, further studies are warranted to validate these findings and define the optimal integration of this treatment approach into clinical practice.

### 4.7. Functional Results and Quality of Life

While TORS presents a less invasive surgical approach for managing HPV+ OPSCC, the literature reveals a notable gap in comparative data on functional outcomes and quality of life across different treatment de-escalation strategies. The present studies, in fact, are characterized by a limited sample size and a relatively short follow-up period. This gap is particularly significant given the typically younger demographic and high survival rates associated with HPV+ OPSCC patients, for whom the long-term quality of life, including swallowing function and other critical functionalities, is a paramount consideration.

TORS is recognized for its minimal invasiveness and potential to yield excellent oncological outcomes. However, the necessity for adjuvant therapy, in some cases, may mitigate TORS’s benefits in terms of functional outcomes, potentially exacerbating morbidity, especially in terms of swallowing function. The nuanced balance between achieving optimal cancer control and preserving quality of life underscores the need for more robust comparative studies. Specifically, more comprehensive data must be collected to compare the functional outcomes and quality of life among patients undergoing TORS and TORS followed by de-escalated adjuvant therapy versus those subjected to de-escalated CRT or standard CRT protocols.

This deficiency in the literature highlights an urgent need for focused research efforts. Such research is essential for guiding clinical decisions that aim for the best oncological outcomes and prioritize patients’ long-term well-being and quality of life.

### 4.8. Further Considerations

In wrapping up our discussion on integrating TORS into clinical settings, it is imperative to critically evaluate the challenges and opportunities that this innovative surgical approach presents. The effective integration of TORS involves a consideration of several key factors.

Surgeon training and proficiency: TORS requires technical insight and a deep understanding of the complex anatomical structures affected by OPSCC. The necessity for robust training programs cannot be overstated; such initiatives ensure that surgeons are well equipped to handle the intricacies of robotic surgery. Moreover, establishing certification processes will uphold the standards of safety and efficacy that are paramount in surgical interventions. This rigorous approach to training will safeguard the quality of care provided to patients and maintain the integrity of the medical profession.

Resource allocation: the financial outlay required for procuring and maintaining robotic systems is substantial. However, one should also consider the potential long-term benefits when evaluating such investments. These include decreased complication rates and shorter recovery periods, which can significantly reduce overall healthcare costs and improve patient throughput. Therefore, a balanced perspective on resource allocation—one that weighs initial costs against long-term savings and patient benefits—is essential for making informed decisions that will benefit healthcare institutions and their patients.

Development of follow-up care protocols: establishing comprehensive follow-up protocols is crucial in monitoring the recovery and assessing the long-term functional outcomes of patients undergoing TORS. Such protocols are indispensable for ensuring ongoing patient health and well-being, evaluating their effectiveness, and refining the practice of TORS. Regular and systematic follow-ups will provide a wealth of data to inform future improvements in technique and patient care protocols.

## 5. Conclusions

TORS can be considered an upfront treatment option for selected patients to de-escalate the management of HPV-related OPSCC. However, the key to its appropriate use lies in carefully selecting candidates based on risk stratification associated with the patient and the tumor. There must be no anatomical or tumor-related contraindications to perform TORS.

In summarizing our review of TORS for de-escalating treatment in HPV-related OPSCC, we recognize the compelling evidence of its benefits. However, we must address several critical gaps through future research to fully leverage TORS in clinical settings.

Long-term clinical outcomes: while the short-term efficacy of TORS is well-documented, long-term survival, recurrence rates, and late complications remain less understood. Ongoing longitudinal studies are crucial to confirm the sustained benefits of TORS and its role in enhancing patient survival over decades.

Comparative effectiveness: there is a conspicuous need for direct comparisons between TORS and conventional non-surgical modalities. More randomized controlled trials could elucidate differential outcomes in efficacy, safety, and quality of life, providing a more robust foundation for treatment decision making.

Integration with emerging therapies: as new treatments such as immunotherapy emerge, their integration with TORS could redefine therapeutic protocols. Investigating these combinations could open up new pathways for personalized medicine, potentially increasing the cure rates while minimizing adverse effects.

By addressing these areas, future research can substantially advance our understanding and application of TORS, ultimately enhancing the therapeutic landscape for patients with HPV-related OPSCC.

## Figures and Tables

**Table 1:** Selected studies.

Author and Year	Country	Type of Study	Sample Size	Robotic System Used	Stages Included	Main Findings	Main Limitations
Achim [8], 2018	USA	Prospective	74	Da Vinci SI	pT1, T2 (7th)	In patients with OPSCC, treatment with TORS alone was associated with improved long-term functional outcomes in swallowing and speech, with QOL metrics returning to near baseline. Patients who received adjuvant treatment did not recover as quickly, and those who underwent CRT in addition to TORS had the greatest risk for poor long-term outcomes.	Wide intervals for follow-up Possible heterogeneity of adjuvant therapy and G tube placement in outside facilities Wide service area and possible differences in accessibility Treatment-specific impact not distinguishable from the effects of different disease burden in the groups
Albergotti [9], 2017	USA	Prospective	51	NS	pT1, T2, T3, TX (7th)	Patients undergoing TORS for OPSCC were prospectively enrolled and their short-term swallowing outcomes were evaluated. Despite elevated EAT-10 scores up to a month post-operatively, adverse dysphagia-related outcomes are rare.	No data on preoperative dysphagia No functional swallowing assessment Trismus and velopharyngeal insufficiency not assessed Patients with complicated postoperative phase not included
Amit [10], 2019	USA	Prospective	86	NS	cT1, T2, T3 (7th)	Symptom burden and QOL improved over time after treatment in low–intermediate-risk OPSCC survivors, regardless of whether primary surgical or nonsurgical treatment was used.	Relatively small sample Possible selection bias Relatively young patients with few comorbidities, influencing generalizability
Baliga [11], 2018	USA	Retrospective	17150	NS	T1, T2 (7th)	Patients with OPSCC treated with TORS had similar survival outcomes to definitive RT, with a lower likelihood of receiving CT.	Selection bias (TORS associated with better performance status) No data on locoregional control, cause-specific survival, and salvage therapies No data on functional outcomes
Biron [12], 2017	Canada	Prospective	47 (18 TORS, 29 mandibulotomies)	Da Vinci S	pT1, T2, T3 (7th)	TORS with radial forearm free flap reconstruction is a safe, effective, and cost-saving alternative to the lip-splitting mandibulotomy approach for the treatment of advanced stage OPSCC.	Data from mandibulotomy group retrospectively collected Only surgical and hospitalization costs evaluated Confounders on length of hospital stay due to progress in postoperative care
Brody [13], 2022	USA	Retrospective	634	NS	pT0, T1, T2, T3, T4 (8th)	Oncologic outcomes of a large HPV+ OPSCC cohort treated with TORS were evaluated to develop a risk prediction model for recurrence. Positive surgical margins were associated with risk for distant metastatic recurrence but not isolated locoregional recurrence.	
Cannavicci [14], 2023	Italy	Retrospective	87	NS	pT0, T1, T2, T3 (8th)	Rates of perioperative complications after TORS were evaluated in a retrospective analysis of 87 consecutive OPSCC patients. No major intraoperative complications and no total local or free flap failure were registered.	
Cannon [15], 2018	USA	Retrospective	88	NS	T1, T2, T3 (7th)	A retrospective case series of 88 patients with OPSCC treated with TORS and simultaneous neck dissection showed excellent survival outcomes with no regional recurrences, suggesting level Ib dissection could be avoided to limit morbidity.	Retrospective and single center, no randomization or standardization No assessment of quality-of-life parameters
Carey [16], 2021	USA	Retrospective	541	NS	pT0, T1, T2, T3, T4 (8th)	LRR rates are low for HPV+ OPSCCs completing TORS and guideline-compliant adjuvant therapy. Patients without indication for adjuvant therapy more often suffer LRR, but these recurrences are generally controllable by salvage therapy.	
Chao [17], 2019	USA	Retrospective	267	NS	pT1, T2, T3, T4a, T4b (8th)	The impact of “package time” from surgery to completion of adjuvant therapy on oncological outcomes in HPV+ OPSCC patients was analyzed. Prolongation of package time appears to compromise locoregional control, but not survival.	Retrospective, single institution Limited number of recurrence events limiting the statistical robustness
Chen [18], 2021 (1)	USA	Retrospective	207	NS	pT0, T1, T2, T3, T4 (8th)	Recurrence and survival in HPV-related OPSCC with single-lymph node metastasis treated with transoral surgery with or without adjuvant therapy were evaluated. Excellent survival with a high rate of successful salvage treatment among patients with regional recurrence was observed.	Retrospective, small sample, some missing data Dual institutional design, with resulting heterogeneity Small number of recurrences and deaths
Chen [19], 2021 (2)	USA	Retrospective	375	NS	pT0, T1, T2, T3, T4 (8th)	The association between smoking, survival, and recurrence in HPV-related OPSCC was retrospectively assessed, showing heavier smoking >20 pack years was strongly associated with worse outcomes.	Retrospective No differentiation of nuances in smoking history No objective smoking measures such as cotinine levels Non-cigarette tobacco exposure and alcohol abuse not considered
Costantino [20], 2023	Korea	Retrospective	198	Da Vinci Si/Xi	cT1, T2, T3, T4a (8th)	In locoregionally advanced OPSCC, neoadjuvant chemotherapy followed by TORS provided excellent tumor control and survival, potentially reducing the need of adjuvant treatment	Retrospective nature, selection bias Relatively short median follow-up time restricting the evaluation of long-term benefits of NCT
Cramer [21], 2018	USA	Retrospective	1677	NS	pT1, T2 (8th)	Deintensification to surgery alone in patients with low- or intermediate-risk features was not associated with a decrease in OS for stage I HPV+ OPSCC. Classic intermediate-risk pathologic features offer diminished prognostic value in HPV+ OPSCC.	Large oncologic registry with potential coding errors No data on recurrence or disease-specific survival Several unmeasured variables Unavailable treatment details
Dhanireddy [22], 2019	USA	Retrospective	219	NS	pT1, T2 (7th)	TORS with directed adjuvant therapy appears to have comparable outcomes to primary CRT in terms of survival and PEG dependence, with no significant differences beyond 12 months.	
De Virgilio [23], 2023	Italy	Retrospective	139	NS	pT0, T1, T2, T3, T4 (8th)	TORS is useful in the management of selected cases of OPSCC to limit the treatment to the surgical approach or to de-intensify adjuvant treatments. The choice of the therapeutic strategy for OPSCC requires evaluation by a multidisciplinary team.	Relatively small number of included patients Retrospective nature
Feng [24], 2022	USA	Retrospective	138	NS	pT0, T1, T2, T3 (8th)	Factors associated with feeding tube placement following TORS for OPSCC were identified in a retrospective series, with a low rate of long-term feeding tube dependence and favorable outcomes.	Practitioner-and patient-related confounders in feeding tube placement Different CT and RT regimens affecting swallowing function
Ford [25], 2014	USA	Retrospective	130	NS	pT1, T2, T3, T4 (7th)	Patients treated for OPSCC with TORS appeared to survive more frequently than those treated with open surgery, suggesting that oncologic outcomes are not sacrificed when using TORS.	Retrospective nature, selection bias Small sample size
Frederiksen [26], 2021	Denmark	Prospective	30	Da Vinci SI HD	pT1, T2 (7th)	A prospective cohort of thirty patients who underwent TORS and neck dissection for early-stage OPSCC showed good long-term oncological outcomes in terms of five-year OS, DSS and RFS.	
Groysman [27], 2022	USA	Retrospective	9267	NS	cT1, T2 (7th)	Socioeconomic and geographic factors are associated with a lower likelihood of patients being treated with TORS or transoral endoscopic surgery.	Retrospective Large oncologic registry with potential coding errors
Haller [28], 2023	USA	Retrospective	2019	NS	pT1, T2, T3, T4 (8th)	The study reports low morbidity and mortality after TORS in clinical trials, examining the safety of de-escalated adjuvant chemoradiotherapy for HPV+ OPSCC.	Retrospective Reporting and selection bias
Hughes [29], 2023	USA	Retrospective	167	NS	pT1, T2, T3 (8th)	Treatment outcomes for patients with early-stage HPV-associated OPSCC appeared comparable between primary TORS or RT, with swallowing dysfunction more frequent in patients requiring more aggressive treatment to the neck.	Sample size Retrospective, selection bias
Isenberg [30], 2020	Denmark	Retrospective	205	Da Vinci	NS	A year-by-year comparative analysis of indications for TORS, hospitalization, and complication rates showed a shift in indications and a reduction in complication rates over time.	
Jackson [31], 2017	USA	Retrospective	105	NS	cT0, T1, T2, T3, T4 (8th)	Matched analysis of patients treated for OPSCC with either TORS or open surgery plus standard-of-care adjuvant therapy showed no difference in disease-specific survival or overall survival with the addition of adjuvant therapy. The risk of gastrostomy tube was higher in those receiving adjuvant therapy.	Retrospective nature Potential bias on treatment preferences Limited sample size because of HPV-related OPSCC largely being typical of younger population
Kaffenberger [32], 2021	USA	Retrospective	73	NS	T1, T2, T3, T4 (7th)	QOL outcomes in advanced-stage, mostly HPV+, OPSCC were assessed through patient-reported outcomes after primary treatment, highlighting the need for continued therapy de-escalation.	Retrospective and cross-sectional design Numerous patients excluded because of lack of data, reducing statistical significance Selection bias in a survivorship clinic
Kucur [33], 2015	USA	Retrospective	73	Da Vinci	cT1, T2, selected T3 (7th)	The utility of TORS in the resection of oropharyngeal cancers extending to PPS was evaluated, showing it to be a safe and feasible technique with minimal complications compared to traditional transcervical techniques.	Relatively small sample size No conventional surgery control group Insufficient long-term data to assess outcomes
Li [34], 2019	USA	Retrospective	2224	NS	cT1, T2 (7th)	Long-term oncologic outcomes and subsequent adjuvant therapy use for patients treated with TORS compared to those treated with TLM and non-robotic surgery were analyzed, showing that TORS patients had equivalent overall survival.	Some variables not captured in the database (risk factors, type of HPV testing, quality of life and complications Focus on overall survival and not cancer-specific survival Possible selection bias
Ling [35], 2016	USA	Retrospective	92	NS	cT0, T1, T2 (7th)	Oncologic outcomes and QOL scores between early-stage OPSCC patients treated with definitive CRT and those treated with TORS ± adjuvant therapy were compared, showing similar rates of control and improved long-term saliva-related QOL for definitive TORS.	Higher disease burden in definitive CRT cohort Small sample size Limited number of completed quality of life surveys Selection bias (only newly diagnosed patients, no patients with recurrent disease)
Lu [36], 2023	USA	Retrospective	255	NS		Swallowing and feeding-tube outcomes in patients with high-risk oropharyngeal cancer treated with trimodality therapy, including TORS and adjuvant chemoradiotherapy, were evaluated, showing low rates of long-term feeding tube dependence and favorable swallowing outcomes.	
Lybak [37], 2017	Norway	Retrospective	232	NS	cT1, Y2, T3, T4 (8th)	OPSCC treatment recommendations and outcomes were compared across two time periods, with a shift from primary RT to surgery and neck dissection, followed by RT. The health-related quality of life scores among successfully treated patients were worse following surgery plus RT than RT only.	The cohort mostly includes patients diagnosed before CRT had replaced RT as main recommended primary treatment, no conclusion can be drawn on the role of chemotherapy
McMullen [38], 2019	USA–Canada	Retrospective	92	NS	pT0, T1, T2 (8th)	Challenges in estimating pathologic nodal metastases and ENE, as well as the need for adjuvant therapy, were analyzed in a multicenter study, indicating that a portion of patients had unanticipated ENE, potentially indicating a need for adjuvant chemotherapy.	Retrospective Multi-institutional nature and interinstitutional variability Limited to radiographic assessment, no implications on adjuvant treatment and outcome prediction
Meccariello [39], 2020	Italy	Retrospective	129	NS	cT0, T1, T2, T3 (surgery group, 8th), cT0, T1, T2, T3, T4, T4a, T4b (definitive CRT group, 8th)	No statistical difference in 5-year survival rate and disease-free interval between TORS and CRT groups in OPSCC patients, with HPV status not affecting the rate of local and regional recurrence.	
Mehanna [40], 2023	UK–Poland	Retrospective	985	NS	cT1, T2, T3, T4 (7th)	Clinical data and samples from a consecutive cohort of 985 OPSCC cases treated with curative intent were collected to develop clinical and/or biomarker predictive models for patient outcome and treatment escalation.	Retrospective nature and lack of randomization Need of external validation in a prospective setting
Nichols [41], 2021	USA	Retrospective	48	NS	pT0, T1, T2 (8th)	The impact of margins, ENE, and adjuvant therapy on survival in 48 patients treated with TORS was assessed, showing high survival rates.	Limited generalizability (almost exclusively Caucasian males with multiple comorbidities, single tertiary center) Small sample size and reduced rate of events Relatively short median follow-up Inability to stratify outcomes based on tumor staging Selection bias
O’Hara [42], 2021	UK	Retrospective	120	Da Vinci	pT0, T1, T2, T3 (8th)	The largest single-centre analysis in the UK reporting oncological outcomes following primary TORS for OPSCC, showing that survival and locoregional control outcomes compare well with other large published series.	Limited ability to draw conclusions on patient and tumor variables and their effect on recurrence Low number of events (recurrence or death) precluding multivariable survival analysis
Olaleye [43], 2023	Australia		102			The TNM-8 AJCC classification was validated in a cohort treated predominantly with primary surgery and adjuvant therapy for HPV-OPSCC, showing comparable survival outcomes.	
Olson [44], 2020	USA	Retrospective	245	NS	pT1, T2 (8th)	The association between sarcopenia and OPSCC survival for patients treated by either primary surgery or definitive RT was characterized, showing improved survival for sarcopenic patients with primary surgical resection.	Several patients were excluded from the analysis owing to a lack of abdominal imaging and this limits the overall power of the study and may introduce a selection bias Not controlled study and the choice of treatment modality was entirely up to the patients and treatment team
Oliver [45], 2022	USA	Retrospective	73661	NS	pT1, T2, T3 (8th)	Data from 73,661 patients with OPSCC treated from 2010 to 2016 were reviewed to investigate the adoption and safety of TORS, demonstrating an increase in TORS utilization and very low risk of severe complications.	The use of NCBD, including possible coding errors and the lack of centralized review by head and neck pathologist The lack of information like smoking history, timing of primary tumor resection, post-operative complications
Park [46], 2017 (1)	Korea	Retrospective	80	NS	pT3, T4 (7th)	TORS-based therapy for stage III–IV OPSCC showed excellent oncological and functional outcomes, with clear margins in the majority of patients and a significant relationship between ENE and recurrence-free survival.	It was a retrospective analysis and not a randomized prospective controlled trial The use of 7th edition of AJCC
Park [47], 2017 (2)	Korea	Clinical trial	31	NS	pT3, T4 (8th)	Neoadjuvant chemotherapy combined with TORS was useful for treating advanced oropharyngeal cancer, showing promising oncologic and functional outcomes.	
Park [48], 2019 (3)	Korea	Retrospective	188	DaVinci	pT1,T2 (8th)	Surgical treatment for p16+ OPSCC patients showed excellent oncologic results, with the AJCC 8th-edition staging system showing a significant relationship with patient survival.	Retrospective analysis of data
Plonowska [49], 2021	USA	Retrospective	95	NS	pTis, T0,T1,T2,T3(8th)	The need for and predictors of nasogastric tube feeding in a cohort of OPSCC patients undergoing TORS were determined, identifying larger tumor size and concurrent bilateral ND as risk factors for NGTF.	The sample sizes may be too small to detect a clinical benefit in this study The duration of NGTF is probably artificially high due to the fact that the swallowing safety assessments have time intervals longer than a week between each Single institution sample composed by patients with early-stage disease The intraoperative placement of NGT may have influenced the recommendations and may distort the results somewhat
Ranta [50], 2021	Finland	Retrospective	263	NS	pT1,T2,T3,T4 (7th)	Long-term QOL was assessed in survivors of OPSCC diagnosed and treated between 2000 and 2009, showing that most survivors reported a good QOL. Single modality treatment group had significantly better QOL outcomes than the combined treatment group.	Treatment modalities were unequally balanced across cancer stages
Rubek [51], 2017	Denmark	Prospective	30	Da Vinci	cT1,T2 (7th)	TORS and concurrent neck dissection were shown to be a safe and feasible procedure for patients with early-stage OPSCC, with a microscopic radical T-site achieved in 97% of patients. However, due to N-site stage migration and ENE, 43% of the patients were eligible for adjuvant therapy.	More than half of the patients in this study were clinically misclassified in their cTNM
Scott [52], 2021 (1)	Denmark	Prospective	44	NS	pT1,T2 (8th)	Patients treated with TORS had high “days alive and out of hospital” (DAOH) in the first 30 and 180 days after treatment, while patients treated with RT had reduced DAOH30 and DAOH180, calling for further large-scale studies.	Patients treated with TORS had a better cTNM-stage compared to patients treated with primary RT. This is a major limitation making comparisons between groups meaningless Selection bias in the RT group and small sample size. Unfortunately, 65/78 eligible RT patients declined to participate
Scott [53], 2021 (2)	Denmark	Prospective	44	NS	cT1,T2 (7th)	Functional and QOL outcomes in the first 12 months after treatment with either primary TORS or primary RT were reviewed, showing overall good functional and QOL outcomes 1 year after treatment for OPSCC regardless of modality.	The small sample size, particularly in the RT group Selection bias because only T1–2 and N0–1 were considered for TORS While all but one eligible TORS patients were included in this study, patients receiving RT were less inclined to participate, as evidenced by the low number of patients enrolled compared to eligible patients in this group possibly skewing the results
Sethia [54], 2018	USA–Turkey	Prospective	111	Da Vinci	pT1,T2,T3,T4 (7th)	Quality of life of OPSCC patients who underwent TORS alone, with adjuvant RT, or adjuvant CRT was prospectively evaluated, showing that TORS alone maintained higher QOL than adjuvant RT or CRT in eating, social function, speech, and overall QOL post-surgery for 6 months.	
Sharma [55], 2016	USA	Retrospective	127	NS	pT1,T2,T3 (7th)	Patients undergoing TORS for OPSCC had statistically indistinguishable survival but lower gastrostomy prevalence compared with patients undergoing nonsurgical therapy for stage-matched OPSCC.	The percentage of patients with unknown p16 status and the difference in the percentage of patients with unknown p16 status between the 2 groups are sources of potential bias The patients undergoing TORS were from a single-study institution Other differences present between the 2 treatment groups included less comorbidity and more non-smokers in the TORS group
Sims [56], 2017	USA	Retrospective	286 total	Da Vinci	pT1,T2,T3,T4 (7th)	Management and oncologic outcomes for patients who developed LRR and distant metastasis DM following TORS for HPV-positive OPSCC were described, showing favorable cancer-specific survival rates and recommending aggressive salvage treatment for appropriate candidates.	Highly selected group of patients who were good candidates for salvage treatment from an oncologic and medical comorbidity standpoint; therefore, selection bias plays a role in the reported survival outcomes Retrospective nature of this study Small overall number of LRRs and DMs Initial undertreatment of some patients with more advanced disease who refused adjuvant therapy Short follow-up
Singh [57], 2023	USA	Retrospective	6301	NS	cT1,T2,T3,T4a (8th)	The National Cancer Database was queried for patients with HPV-associated OPSCCs initially managed with surgery with intermediate risk factors (IRFs) or high-risk factors (HRFs), showing that IRFs should continue to be utilized to guide decisions on receipt of adjuvant therapy and suggesting potential opportunities for the de-escalation of therapy among patients with HRFs.	Unable to assess toxicity between PORT and POCRT arms Lack of information on either LRC, relapse-free survival, or distant metastasis rates Lack of information on the dose utilized, number of cycles received the specific chemotherapy utilized, or need for dose reductions during receipt of POCRT Lack of information on smoking/pack–year history to risk stratify patients into low risk vs. intermediate risk, which has been utilized in previous and currently accruing de-escalation trials to determine eligibility for enrollment Lack of information on how close margins were, which is regarded as an IRF, or whether patients with positive margins had a re-resection High risk of inaccurate and variable coding of data regarding IRFs and/or HRFs in addition to other relevant factors, particularly with respect to final margin status
Sinha [58], 2015	USA	Prospective	220		pT1,T2,T3,T4 (7th)	A prospectively assembled cohort of transoral surgery + neck dissection ± adjuvant therapy-treated p16+ OPSCC patients was analyzed, identifying ≥5 nodes and T3-T4 classification as predictors for recurrence and prognostic for DSS.	A comparative analysis of prognostication between p16+ and p16-negative OPSCC cannot be made from the dataset we present Inability to analyze pathologic node level data
Stephens [59], 2023	USA	Prospective	48	NS	pT1,T2,T3 (8th)	Changes in patient-reported outcomes measures on QOL in relation to pre-surgery QOL in patients who underwent surgery alone for early-stage HPV+ OPSCC were evaluated, showing favorable QOL outcomes.	The study cohort was treated at a tertiary care center, where patients have access to an interdisciplinary team of speech language pathologists and physical and occupational therapists, which may have contributed to improved QOL outcomes when compared to a broader cohort There is a bias in data collection due to the fact that not all patients adhered to preoperative assessments and some did not complete follow-up
Sun [60], 2021	USA	Retrospective	178	NS	cT1,T2,T3,T4 (7th)	A cohort of 178 consecutive patients with HPV+ OPSCC receiving TORS + trimodality therapy (TMT) was analyzed, showing a 5-year survival of 93.6% and low rates of long-term feeding tube dependence.	Impossibility to accurately assess rates of nausea, dysphagia, dysgeusia, and mucositis, and report validated functional outcomes data There are many differences between patients treated with noncisplatin chemotherapy and cisplatin-chemotherapy, in terms of number, age, functional status, and indications for therapy: all of these factors limit any survival comparisons between cisplatin- and noncisplatin-treated cohorts
Swisher-McClure [61], 2020	USA	Prospective	60	NS	pT1,T2 (7th)	Sixty patients underwent de-intensified RT approach after initial surgical resection for HPV-associated OPSCC, with a 2-year local control of 98.3% and OS of 100% at the time of analysis.	The trial enrolled a relatively small number of patients and current follow-up of 2.4 years The non-randomized study precludes direct comparison and definitive conclusions regarding comparative toxicity All patients had treatment at a single institution and surgeries were performed by highly experienced TORS surgeons which may limit generalizability
Van Abel [62], 2019 (1)	USA	Retrospective	267	NS	pT1,T2,T3,T4 (7th) and pT1,T2,T3,T4a (8th)	Clinical data of 267 patients who underwent TORS ± standard adjuvant therapy were recorded, reviewing swallowing, airway, and speech outcomes, with a small percentage of patients remaining PEG-dependent at last follow-up.	Selection bias into treatment arms Impossibility to compare QOL outcomes directly to TORS The timing of post-adjuvant formal evaluations was limited for most patients to <12 months, making a comment on the long-term recovery of function impossible The adjuvant therapy was frequently sought closer to home, making it challenging to assess the quality of adjuvant therapy
Van Abel [63], 2020 (2)	USA	Retrospective	78	Da Vinci	pT1,T2,T3,T4 (8th)	A retrospective cohort study included 78 consecutive patients undergoing TORS procedures with da Vinci single-port robot, comparing outcomes to a historical cohort of OPSCC patients, showing no significant differences in operative time or post-TORS bleeds.	It is retrospective in design, and the sample size is small In comparing the SP and Si cohorts, also recognize the potential for confusion bias other epidemiological or practice patterns that may affect surgical outcomes over a broad range of time SP patients were significantly older than Si patients, which likely reflect the aging epidemiology of OPSCC Single-center study
Van Loon [64], 2015	The Netherlands	Retrospective	18	NS	pT1,T2 (7th)	TORS appears to be a safe treatment for early-stage T1-2 N0 OPSCC, with successful tumor removal with clear margins in most patients. However, careful patient selection is crucial to avoid the need for adjuvant radiotherapy.	Because of the absence of baseline measurements, it was not possible to analyze whether the health-related problems returned to pretreatment levels at 12 months
Waltonen [65], 2022	USA	Retrospective	74	NS	pT1,T2,T3 (8th)	Seventy-four patients who underwent surgery for OPSCC and were not recommended to adjuvant therapy or declined it had a low risk of recurrence, with lymphovascular invasion and lymph node features such as N-stage and presence of ENE having a statistically significant impact on relapse.	The retrospective nature of this single-institution study has inherent biases The small sample size Functional outcomes of these patients are not included in this paper
Wright [66], 2021	USA	Retrospective	676	NS	pT1,T2,T3 (8th)	The prognostic significance of oligometastatic versus polymetastatic disease in HPV-associated OPSCC and the impact of definitive tumor-directed therapy on survival outcomes were assessed, showing improved median OS) for oligometastatic patients.	Retrospective design of this study The heterogeneity introduces the possibility of selection bias and unmeasured confounding For the multivariable analysis in this study, the sample size is small for our analyses of metastatic disease outcomes and the MVA model should be interpreted with limited patient numbers in mind
Xu [67], 2020	USA	Retrospective	76	NS	pT1,T2 (8th)	Long-term post-treatment overall QOL was comparably high for most AJCC eighth edition early-stage HPV+ OPSCC patients, with nuances specific to treatment modality.	Retrospective QOL studies including a small sample size, with even smaller subset groups, and potential bias in self-selection Compromised by the 66% response rate among eligible participants Without randomized treatment, factors influencing tge decision of treatment modality are not adequately captured
Yin [68], 2020	China	Retrospective	294	NS	cT1,T2,T3 (8th)	The QOL of HPV-related OPSCC patients was measured before and 3–6 months after definitive treatment, demonstrating that the treatment decreased the QOL of these patients.	Relatively small sample size There still were some hidden predictor variables that are not involved in this research, such as comorbidities and a history of illicit drug used Side effects of radiation therapy, that are observed quite late in follow-up period, should have been tested in the current study for precautions to avoid or ease the side effects
Zebolsky [69], 2021	USA	Retrospective	136	NS	cT1,T2 (7th)	This study evaluated the risk of adverse histopathologic features in patients with HPV-positive OPSCC selected for primary surgery, showing about one-quarter will have pathologic ENE and/or PSM, indications for adjuvant CRT.	Single-institution study, limited sample size, and event rates prevent the authors from making definitive conclusions Selection for surgery was determined by a combination of multidisciplinary tumor board consensus, patient-surgeon discussions, and patient preference, leading to decisions and outcomes that may not be generalizable to other institutions The histopathologic analyses were performed by multiple pathologists, generating the possibility of interrater discrepancies on the interpretation and grading of pathologic ENE
Zubair [70], 2021	UK	Retrospective	272	NS	cT1,T2,T3,T4 (8th)	The 8th edition of the UICC TNM staging rules for OPSCC based on HPV tumor status was validated in 272 patients, with a dichotomous disease biology confirmed. Patients with HPV-positive T1 and T2 primary tumors have an excellent prognosis when treated with non-surgical treatment regimens.	
